# 3-(4-Fluoro­phenyl­sulfin­yl)-2,5,7-trimethyl-1-benzofuran

**DOI:** 10.1107/S160053681000293X

**Published:** 2010-01-30

**Authors:** Hong Dae Choi, Pil Ja Seo, Byeng Wha Son, Uk Lee

**Affiliations:** a Department of Chemistry, Dongeui University, San 24 Kaya-dong Busanjin-gu, Busan 614-714, Republic of Korea; b Department of Chemistry, Pukyong National University, 599-1 Daeyeon 3-dong, Nam-gu, Busan 608-737, Republic of Korea

## Abstract

In the title mol­ecule, C_17_H_15_FO_2_S, the O atom and the 4-fluoro­phenyl group of the 4-fluoro­phenyl­sulfinyl substituent lie on opposite sides of the benzofuran fragment. The mean planes of the benzofuran and 4-fluoro­phenyl fragments form a dihedral angle of 86.07 (4)°. In the crystal structure, weak inter­molecular C—H⋯O hydrogen bonds link the mol­ecules into centrosymmetric dimers, which are further linked *via* inter­molecular C—H⋯π inter­actions.

## Related literature

For the crystal structures of similar 2-methyl-3-phenyl­sulfinyl-1-benzofuran derivatives, see: Choi *et al.* (2007 ▶, 2008**a* ▶,b* ▶). For the biological activity of benzofuran compounds, see: Aslam *et al.* (2006 ▶); Galal *et al.* (2009 ▶); Khan *et al.* (2005 ▶). For natural products with benzofuran rings, see: Akgul & Anil (2003 ▶); Soekamto *et al.* (2003 ▶).
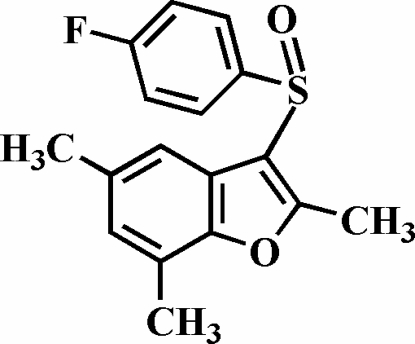


## Experimental

### 

#### Crystal data

C_17_H_15_FO_2_S
*M* *_r_* = 302.35
Triclinic, 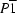

*a* = 6.2558 (2) Å
*b* = 11.1848 (3) Å
*c* = 11.9766 (5) Å
α = 110.355 (2)°
β = 99.448 (2)°
γ = 104.130 (1)°
*V* = 732.75 (4) Å^3^
*Z* = 2
Mo *K*α radiation
μ = 0.23 mm^−1^
*T* = 172 K
0.43 × 0.28 × 0.26 mm

#### Data collection

Bruker SMART APEXII CCD diffractometer
Absorption correction: multi-scan (*SADABS*; Bruker, 2009 ▶) *T*_min_ = 0.681, *T*_max_ = 0.746
12781 measured reflections
3350 independent reflections
3071 reflections with *I* > 2σ(*I*)
*R*_int_ = 0.026

#### Refinement

*R*[*F* ^2^ > 2σ(*F* ^2^)] = 0.036
*wR*(*F* ^2^) = 0.102
*S* = 1.03
3350 reflections
193 parameters
H-atom parameters constrained
Δρ_max_ = 0.27 e Å^−3^
Δρ_min_ = −0.37 e Å^−3^

### 

Data collection: *APEX2* (Bruker, 2009 ▶); cell refinement: *SAINT* (Bruker, 2009 ▶); data reduction: *SAINT*; program(s) used to solve structure: *SHELXS97* (Sheldrick, 2008 ▶); program(s) used to refine structure: *SHELXL97* (Sheldrick, 2008 ▶); molecular graphics: *ORTEP-3* (Farrugia, 1997 ▶) and *DIAMOND* (Brandenburg, 1998 ▶); software used to prepare material for publication: *SHELXL97*.

## Supplementary Material

Crystal structure: contains datablocks global, I. DOI: 10.1107/S160053681000293X/cv2691sup1.cif

Structure factors: contains datablocks I. DOI: 10.1107/S160053681000293X/cv2691Isup2.hkl

Additional supplementary materials:  crystallographic information; 3D view; checkCIF report

## Figures and Tables

**Table 1:** Hydrogen-bond geometry (Å, °) *Cg* is the centroid of the C2–C7 ring.

*D*—H⋯*A*	*D*—H	H⋯*A*	*D*⋯*A*	*D*—H⋯*A*
C13—H13⋯O2^i^	0.95	2.50	3.194 (2)	130
C11—H11*B*⋯*Cg*^ii^	0.98	2.87	3.663 (2)	139

Symmetry codes: (i) 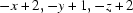; (ii) 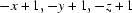.

**Fig. 1.**
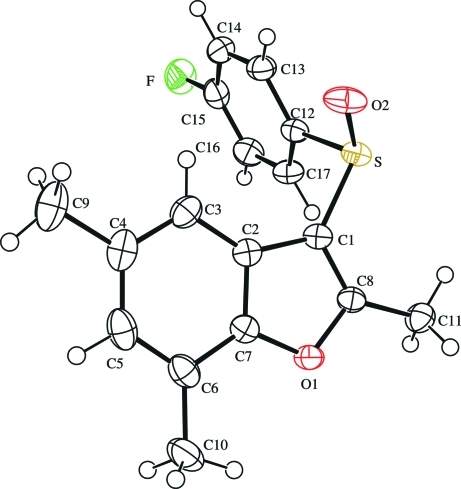
The molecular structure of the title compound with the atom numbering scheme. Displacement ellipsoids are drawn at the 50 % probability level. H atoms are presented as a small spheres of arbitrary radius.

**Fig. 2.**
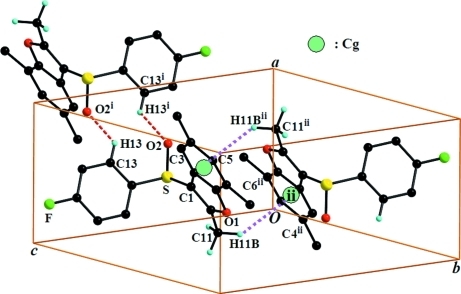
C–H···O and C–H···π interactions (dotted lines) in the crystal structure of the title compound. Cg denotes the ring (C2-C7) centroid. [Symmetry codes: (i) - x + 2, - y + 1, - z + 2; (ii) - x + 1, - y + 1, - z + 1.]

## supplementary crystallographic information

### Comment

Molecules containing benzofuran skeleton show potent biological activities such as antifungal (Aslam *et al.*, 2006), antitumor and antiviral (Galal *et al.*, 2009), antimicrobial (Khan *et al.*, 2005) properties. These compounds are widely occurring in nature (Akgul & Anil, 2003; Soekamto *et al.* 2003). As a part of our ongoing studies of the effect of side chain substituents on the solid state structures of 2-methyl-3-phenylsulfinyl-1-benzofuran analogues (Choi *et al.*, 2007, 2008*a,b*), we report the crystal structure of the title compound (Fig. 1).

The benzofuran unit is essentially planar, with a mean deviation of 0.007 (1) Å from the least-squares plane defined by the nine constituent atoms. The 4-fluorophenyl ring is almost perpendicular to the plane of the benzofuran fragment [86.07 (4)°] and is tilted slightly towards it. The crystal packing (Fig. 2) is stabilized by a weak intermolecular C–H···O hydrogen bond between the 4-fluorophenyl H atom and the oxygen of the S═O unit (Table 1). The molecular packing (Fig. 2) is further stabilized by an intermolecular C–H···π interaction between the methyl H atom and the benzene ring of an adjacent benzofuran system, with a C11–H11B···Cg^ii^ (Table 1; Cg is the centroid of the C2-C7 benzene ring).

### Experimental

77% 3-Chloroperoxybenzoic acid (291 mg, 1.3 mmol) was added in small portions to a stirred solution of 3-(4-fluorophenylsulfanyl)-2,5,7-trimethyl-1-benzofuran (343 mg, 1.2 mmol) in dichloromethane (30 mL) at 273 K. After being stirred at room temperature for 3h, the mixture was washed with saturated sodium bicarbonate solution and the organic layer was separated, dried over magnesium sulfate, filtered and concentrated in vacuum. The residue was purified by column chromatography (hexane-ethyl acetate, 1:1 v/v) to afford the title compound as a colorless solid [yield 82%, m.p. 433-434 K; *R*_f_ = 0.65 (hexane-ethyl acetate, 1:1 v/v)]. Single crystals suitable for X-ray diffraction were prepared by slow evaporation of a solution of the title compound in ethyl acetate at room temperature.

### Refinement

All H atoms were positioned geometrically and refined using a riding model, with C–H = 0.95 Å for aryl and 0.98 Å for methyl H atoms. *U*_iso_(H) = 1.2*U*_eq_(C) for aryl and 1.5*U*_eq_(C) for methyl H atoms.

### Crystal data

**Table:**

C_17_H_15_FO_2_S	*Z* = 2
*M**_r_* = 302.35	*F*(000) = 316
Triclinic, *P*1	*D*_x_ = 1.370 Mg m^−^^3^
Hall symbol: -P 1	Mo *K*α radiation, λ = 0.71073 Å
*a* = 6.2558 (2) Å	Cell parameters from 9234 reflections
*b* = 11.1848 (3) Å	θ = 3.3–27.5°
*c* = 11.9766 (5) Å	µ = 0.23 mm^−^^1^
α = 110.355 (2)°	*T* = 172 K
β = 99.448 (2)°	Block, colourless
γ = 104.130 (1)°	0.43 × 0.28 × 0.26 mm
*V* = 732.75 (4) Å^3^	

### Data collection

**Table:**

Bruker SMART APEXII CCD diffractometer	3350 independent reflections
Radiation source: Rotating Anode	3071 reflections with *I* > 2σ(*I*)
Bruker HELIOS graded multilayer optics	*R*_int_ = 0.026
Detector resolution: 10.0 pixels mm^-1^	θ_max_ = 27.5°, θ_min_ = 1.9°
φ and ω scans	*h* = −8→8
Absorption correction: multi-scan (*SADABS*; Bruker, 2009)	*k* = −14→14
*T*_min_ = 0.681, *T*_max_ = 0.746	*l* = −15→15
12781 measured reflections	

### Refinement

**Table:**

Refinement on *F*^2^	Primary atom site location: structure-invariant direct methods
Least-squares matrix: full	Secondary atom site location: difference Fourier map
*R*[*F*^2^ > 2σ(*F*^2^)] = 0.036	Hydrogen site location: difference Fourier map
*wR*(*F*^2^) = 0.102	H-atom parameters constrained
*S* = 1.03	*w* = 1/[σ^2^(*F*_o_^2^) + (0.0539*P*)^2^ + 0.3002*P*] where *P* = (*F*_o_^2^ + 2*F*_c_^2^)/3
3350 reflections	(Δ/σ)_max_ < 0.001
193 parameters	Δρ_max_ = 0.27 e Å^−^^3^
0 restraints	Δρ_min_ = −0.37 e Å^−^^3^

### Special details

**Table:**

Geometry. All esds (except the esd in the dihedral angle between two l.s. planes) are estimated using the full covariance matrix. The cell esds are taken into account individually in the estimation of esds in distances, angles and torsion angles; correlations between esds in cell parameters are only used when they are defined by crystal symmetry. An approximate (isotropic) treatment of cell esds is used for estimating esds involving l.s. planes.
Refinement. Refinement of F^2^ against ALL reflections. The weighted R-factor wR and goodness of fit S are based on F^2^, conventional R-factors R are based on F, with F set to zero for negative F^2^. The threshold expression of F^2^ > 2sigma(F^2^) is used only for calculating R-factors(gt) etc. and is not relevant to the choice of reflections for refinement. R-factors based on F^2^ are statistically about twice as large as those based on F, and R- factors based on ALL data will be even larger.

### Fractional atomic coordinates and isotropic or equivalent isotropic displacement parameters (Å^2^)

**Table:**

	*x*	*y*	*z*	*U*_iso_*/*U*_eq_	
S	0.61072 (6)	0.50406 (3)	0.83633 (3)	0.02837 (11)	
F	0.28727 (19)	0.04833 (10)	0.97162 (10)	0.0498 (3)	
O1	0.23445 (16)	0.38938 (10)	0.49810 (8)	0.0282 (2)	
O2	0.86249 (18)	0.53145 (12)	0.85499 (10)	0.0409 (3)	
C1	0.4760 (2)	0.42813 (13)	0.67695 (12)	0.0262 (3)	
C2	0.5133 (2)	0.32114 (13)	0.58028 (12)	0.0265 (3)	
C3	0.6563 (3)	0.24226 (15)	0.57323 (14)	0.0329 (3)	
H3	0.7644	0.2546	0.6454	0.040*	
C4	0.6370 (3)	0.14528 (15)	0.45835 (16)	0.0380 (3)	
C5	0.4753 (3)	0.12862 (15)	0.35294 (15)	0.0391 (4)	
H5	0.4639	0.0608	0.2755	0.047*	
C6	0.3315 (3)	0.20587 (14)	0.35596 (13)	0.0337 (3)	
C7	0.3589 (2)	0.30150 (13)	0.47270 (12)	0.0273 (3)	
C8	0.3107 (2)	0.46565 (13)	0.62320 (11)	0.0261 (3)	
C9	0.7900 (3)	0.05875 (18)	0.4476 (2)	0.0522 (5)	
H9A	0.9355	0.1056	0.4379	0.078*	
H9B	0.7145	−0.0271	0.3754	0.078*	
H9C	0.8196	0.0416	0.5226	0.078*	
C10	0.1578 (3)	0.19014 (17)	0.24463 (14)	0.0464 (4)	
H10A	0.0033	0.1588	0.2532	0.070*	
H10B	0.1734	0.1242	0.1698	0.070*	
H10C	0.1834	0.2772	0.2382	0.070*	
C11	0.1968 (3)	0.56646 (15)	0.67177 (13)	0.0337 (3)	
H11A	0.0400	0.5202	0.6690	0.051*	
H11B	0.1925	0.6186	0.6210	0.051*	
H11C	0.2823	0.6274	0.7575	0.051*	
C12	0.5089 (2)	0.35932 (13)	0.87080 (11)	0.0264 (3)	
C13	0.6686 (2)	0.31841 (15)	0.92889 (12)	0.0310 (3)	
H13	0.8279	0.3625	0.9454	0.037*	
C14	0.5933 (3)	0.21171 (15)	0.96292 (13)	0.0350 (3)	
H14	0.6994	0.1810	1.0023	0.042*	
C15	0.3616 (3)	0.15211 (14)	0.93807 (13)	0.0348 (3)	
C16	0.1997 (3)	0.19321 (15)	0.88255 (14)	0.0361 (3)	
H16	0.0407	0.1499	0.8678	0.043*	
C17	0.2753 (2)	0.29939 (15)	0.84890 (13)	0.0326 (3)	
H17	0.1683	0.3309	0.8112	0.039*	

### Atomic displacement parameters (Å^2^)

**Table:**

	*U*^11^	*U*^22^	*U*^33^	*U*^12^	*U*^13^	*U*^23^
S	0.02613 (19)	0.03171 (19)	0.02281 (17)	0.00535 (14)	0.00233 (13)	0.01064 (13)
F	0.0627 (7)	0.0372 (5)	0.0530 (6)	0.0114 (5)	0.0166 (5)	0.0253 (4)
O1	0.0271 (5)	0.0333 (5)	0.0240 (4)	0.0108 (4)	0.0038 (4)	0.0120 (4)
O2	0.0236 (5)	0.0554 (7)	0.0374 (5)	0.0007 (5)	0.0002 (4)	0.0234 (5)
C1	0.0236 (6)	0.0306 (6)	0.0240 (6)	0.0080 (5)	0.0055 (5)	0.0117 (5)
C2	0.0237 (6)	0.0285 (6)	0.0278 (6)	0.0069 (5)	0.0080 (5)	0.0129 (5)
C3	0.0284 (7)	0.0352 (7)	0.0417 (8)	0.0131 (6)	0.0124 (6)	0.0200 (6)
C4	0.0403 (8)	0.0319 (7)	0.0522 (9)	0.0148 (6)	0.0261 (7)	0.0208 (7)
C5	0.0491 (9)	0.0294 (7)	0.0377 (7)	0.0090 (6)	0.0233 (7)	0.0098 (6)
C6	0.0389 (8)	0.0304 (7)	0.0275 (6)	0.0044 (6)	0.0116 (6)	0.0103 (5)
C7	0.0269 (7)	0.0285 (6)	0.0269 (6)	0.0073 (5)	0.0084 (5)	0.0122 (5)
C8	0.0246 (6)	0.0298 (6)	0.0241 (6)	0.0075 (5)	0.0063 (5)	0.0121 (5)
C9	0.0561 (11)	0.0433 (9)	0.0754 (13)	0.0285 (8)	0.0380 (10)	0.0278 (9)
C10	0.0601 (11)	0.0419 (8)	0.0245 (7)	0.0067 (8)	0.0047 (7)	0.0086 (6)
C11	0.0315 (7)	0.0368 (7)	0.0357 (7)	0.0160 (6)	0.0099 (6)	0.0145 (6)
C12	0.0257 (6)	0.0304 (6)	0.0210 (5)	0.0083 (5)	0.0049 (5)	0.0092 (5)
C13	0.0264 (7)	0.0374 (7)	0.0280 (6)	0.0119 (6)	0.0053 (5)	0.0118 (5)
C14	0.0393 (8)	0.0368 (7)	0.0314 (7)	0.0187 (6)	0.0065 (6)	0.0138 (6)
C15	0.0454 (9)	0.0280 (6)	0.0294 (6)	0.0100 (6)	0.0120 (6)	0.0105 (5)
C16	0.0296 (7)	0.0377 (7)	0.0359 (7)	0.0047 (6)	0.0078 (6)	0.0138 (6)
C17	0.0257 (7)	0.0396 (7)	0.0316 (7)	0.0100 (6)	0.0041 (5)	0.0154 (6)

### Geometric parameters (Å, °)

**Table:**

S—O2	1.4919 (11)	C9—H9A	0.9800
S—C1	1.7521 (13)	C9—H9B	0.9800
S—C12	1.8013 (14)	C9—H9C	0.9800
F—C15	1.3591 (17)	C10—H10A	0.9800
O1—C8	1.3702 (15)	C10—H10B	0.9800
O1—C7	1.3814 (16)	C10—H10C	0.9800
C1—C8	1.3567 (19)	C11—H11A	0.9800
C1—C2	1.4450 (18)	C11—H11B	0.9800
C2—C7	1.3899 (19)	C11—H11C	0.9800
C2—C3	1.3942 (19)	C12—C13	1.3811 (19)
C3—C4	1.388 (2)	C12—C17	1.3885 (19)
C3—H3	0.9500	C13—C14	1.393 (2)
C4—C5	1.406 (2)	C13—H13	0.9500
C4—C9	1.509 (2)	C14—C15	1.372 (2)
C5—C6	1.387 (2)	C14—H14	0.9500
C5—H5	0.9500	C15—C16	1.378 (2)
C6—C7	1.3856 (19)	C16—C17	1.385 (2)
C6—C10	1.503 (2)	C16—H16	0.9500
C8—C11	1.4799 (19)	C17—H17	0.9500
			
O2—S—C1	108.53 (6)	H9A—C9—H9C	109.5
O2—S—C12	105.94 (6)	H9B—C9—H9C	109.5
C1—S—C12	97.96 (6)	C6—C10—H10A	109.5
C8—O1—C7	106.44 (10)	C6—C10—H10B	109.5
C8—C1—C2	107.66 (11)	H10A—C10—H10B	109.5
C8—C1—S	122.90 (10)	C6—C10—H10C	109.5
C2—C1—S	129.43 (10)	H10A—C10—H10C	109.5
C7—C2—C3	119.26 (13)	H10B—C10—H10C	109.5
C7—C2—C1	104.44 (11)	C8—C11—H11A	109.5
C3—C2—C1	136.30 (13)	C8—C11—H11B	109.5
C4—C3—C2	118.46 (14)	H11A—C11—H11B	109.5
C4—C3—H3	120.8	C8—C11—H11C	109.5
C2—C3—H3	120.8	H11A—C11—H11C	109.5
C3—C4—C5	119.76 (14)	H11B—C11—H11C	109.5
C3—C4—C9	119.71 (16)	C13—C12—C17	121.61 (13)
C5—C4—C9	120.53 (15)	C13—C12—S	118.26 (11)
C6—C5—C4	123.51 (14)	C17—C12—S	119.88 (11)
C6—C5—H5	118.2	C12—C13—C14	119.19 (13)
C4—C5—H5	118.2	C12—C13—H13	120.4
C7—C6—C5	114.30 (14)	C14—C13—H13	120.4
C7—C6—C10	121.21 (15)	C15—C14—C13	118.22 (14)
C5—C6—C10	124.49 (14)	C15—C14—H14	120.9
O1—C7—C6	124.51 (13)	C13—C14—H14	120.9
O1—C7—C2	110.78 (11)	F—C15—C14	118.43 (14)
C6—C7—C2	124.70 (13)	F—C15—C16	118.07 (14)
C1—C8—O1	110.67 (11)	C14—C15—C16	123.49 (14)
C1—C8—C11	133.59 (12)	C15—C16—C17	118.13 (14)
O1—C8—C11	115.72 (11)	C15—C16—H16	120.9
C4—C9—H9A	109.5	C17—C16—H16	120.9
C4—C9—H9B	109.5	C16—C17—C12	119.34 (14)
H9A—C9—H9B	109.5	C16—C17—H17	120.3
C4—C9—H9C	109.5	C12—C17—H17	120.3
			
O2—S—C1—C8	−134.93 (12)	C1—C2—C7—O1	0.50 (14)
C12—S—C1—C8	115.24 (12)	C3—C2—C7—C6	1.2 (2)
O2—S—C1—C2	44.44 (14)	C1—C2—C7—C6	−178.86 (13)
C12—S—C1—C2	−65.40 (13)	C2—C1—C8—O1	0.87 (15)
C8—C1—C2—C7	−0.82 (15)	S—C1—C8—O1	−179.65 (9)
S—C1—C2—C7	179.74 (11)	C2—C1—C8—C11	179.02 (14)
C8—C1—C2—C3	179.13 (15)	S—C1—C8—C11	−1.5 (2)
S—C1—C2—C3	−0.3 (2)	C7—O1—C8—C1	−0.55 (15)
C7—C2—C3—C4	−0.7 (2)	C7—O1—C8—C11	−179.07 (11)
C1—C2—C3—C4	179.31 (15)	O2—S—C12—C13	12.66 (12)
C2—C3—C4—C5	−0.2 (2)	C1—S—C12—C13	124.61 (11)
C2—C3—C4—C9	179.74 (13)	O2—S—C12—C17	−172.98 (11)
C3—C4—C5—C6	0.8 (2)	C1—S—C12—C17	−61.03 (12)
C9—C4—C5—C6	−179.12 (14)	C17—C12—C13—C14	2.0 (2)
C4—C5—C6—C7	−0.4 (2)	S—C12—C13—C14	176.26 (10)
C4—C5—C6—C10	179.89 (15)	C12—C13—C14—C15	−0.6 (2)
C8—O1—C7—C6	179.37 (13)	C13—C14—C15—F	−179.71 (12)
C8—O1—C7—C2	0.00 (14)	C13—C14—C15—C16	−0.8 (2)
C5—C6—C7—O1	−179.85 (12)	F—C15—C16—C17	179.68 (13)
C10—C6—C7—O1	−0.2 (2)	C14—C15—C16—C17	0.7 (2)
C5—C6—C7—C2	−0.6 (2)	C15—C16—C17—C12	0.7 (2)
C10—C6—C7—C2	179.13 (14)	C13—C12—C17—C16	−2.0 (2)
C3—C2—C7—O1	−179.46 (12)	S—C12—C17—C16	−176.20 (11)

### Hydrogen-bond geometry (Å, °)

**Table:**

Cg is the centroid of the C2–C7 ring.

**Table:**

*D*—H···*A*	*D*—H	H···*A*	*D*···*A*	*D*—H···*A*
C13—H13···O2^i^	0.95	2.50	3.194 (2)	130
C11—H11B···Cg^ii^	0.98	2.87	3.663 (2)	139

Symmetry codes: (i) −*x*+2, −*y*+1, −*z*+2; (ii) −*x*+1, −*y*+1, −*z*+1.
